# CdS Dots, Rods and Platelets—How to Obtain Predefined Shapes in a One-Pot Synthesis of Nanoparticles

**DOI:** 10.3390/ma14030476

**Published:** 2021-01-20

**Authors:** Hanna Woznica, Mateusz Banski, Artur Podhorodecki

**Affiliations:** Department of Experimental Physics, Wroclaw University of Science and Technology, Wybrzeze Wyspianskiego 27, 50-370 Wroclaw, Poland; hanna.woznica@pwr.edu.pl (H.W.); artur.p.podhorodecki@pwr.edu.pl (A.P.)

**Keywords:** nanoplatelets, CdS, quantum dots, semiconductors, zinc acetate

## Abstract

In recent years, numerous protocols for nanoplatelet synthesis have been developed. Here, we present a facile, one-pot method for controlling cadmium sulfide (CdS) nanoparticles’ shape that allows for obtaining zero-dimensional, one-dimensional, or two-dimensional structures. The proposed synthesis protocol is a simple heating-up approach and does not involve any inconvenient steps such as injection and/or pouring the precursors at elevated temperatures. Because of this, the synthesis protocol is highly repeatable. A gradual increase in the zinc acetate concentration causes the particles’ shape to undergo a transition from isotropic quantum dots through rods to highly anisotropic nanoplatelets. We identified conditions at which synthesized platelets were purely five monolayers thick. All samples acquired during different stages of the reaction were characterized via optical spectroscopy, which allowed for the identification of the presence of high-temperature, magic-size clusters prior to the platelets’ formation.

## 1. Introduction

Colloidal quantum dots (QDs) have been extensively studied for many years since they show unique qualities present only in the quantum regime. The ability to control the size and size distribution is critical in the wet-chemistry synthetic approach as it determines the optical properties such as the emission wavelength and peak broadening. With the development of synthesis technology, an increasing number of ways of manipulating crystals’ geometry at the nanoscale has emerged. Nowadays, not only are we able to precisely choose the size but also the shape of the crystallites. Different morphologies ranging from zero-dimensional (0D) spheres, cubes and polyhedrons [1,2,3] to one-dimensional (1D) rods and wires [4,5] as well as two-dimensional (2D) platelets [6] have been described in the literature. There are many different mechanisms behind the ability to control shape [7]. In general, crystal energy always tends to a minimum in given conditions; therefore, the shape is determined by the surface free energy of each facet. These energies can be manipulated, e.g., by adding specific ligands to the synthesis solution. The ligands interact differently with each facet depending on their structure and atomic composition and, thus, growth in a chosen direction may be suppressed, leading to anisotropic shapes [8,9].

Zero dimensional and 1D structures have been studied for a long time, and in recent years, cadmium-based colloidal nanoplatelets (NPLs)—as members of the 2D family—have also gained popularity due to the fact of their extremely narrow photoluminescence features with full width at half maximum (FWHM) as low as several nanometers [6]. Moreover, it was demonstrated that NPLs are suitable for applications such as light emitting diodes [10,11] or laser diodes [12,13,14,15,16,17]. In these applications NPLs maintain a narrow and precise bandwidth; thus, they enable the exceptional color purity.

The mechanism of NPL formation from II–VI semiconducting materials was a subject of discussion for a long time. Wang et al. [18,19] suggested that wurtzite nanoplatelets—or as they called them, quantum platelets (QPs)—form in lamellar mesophases, and that the intermediate stadium of platelet growth are magic size clusters (MSCs), clusters comprising a strictly defined number of atoms, whereas Riedinger et al. [20] stated that molecular templates are not indispensable for achieving a 2D shape. They have shown that in the case of zinc blende platelets, the critical parameter is the cadmium precursor solubility. When the precursor is poorly soluble in the reaction mixture, phase separation occurs, and platelets can form in isolated droplets. Furthermore, the addition of short ligand-long ligand composition, which is usually used in NPL synthesis (e.g., propionic acid and oleic acid [20]), is not required, and the platelets can even grow in the absence of a solvent. The group also presented a model of 2D growth that assumes that in specific reaction conditions the crystal growth is not limited by diffusion of monomers to the crystal surface but by heterogeneous island nucleation. As the heterogeneous nucleation barrier is lower for narrow facets than for wide facets of the growing crystal, lateral growth is favored, and highly anisotropic shapes can emerge even for isotropic crystal structures.

**Figure 1:**
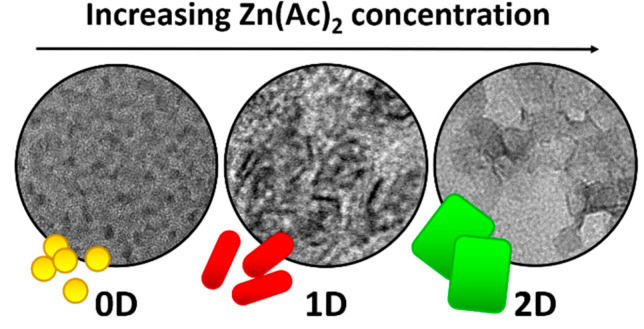
Schematic illustration of the shape control method: increasing the amount of Zn(Ac)_2_ reagent in the initial synthesis solution causes the transition from 0D to 1D and further to 2D nanocrystals.

In this work, we studied the growth of cadmium sulfide (CdS) NPLs in one-pot synthesis with the use of zinc acetate (Zn(Ac)_2_) as a shape-defining reagent. We attempted to make our synthesis procedure as simple as possible in response to the issues with existing protocols. Very often the recipes presented in the literature are hard or even impossible to reproduce, because they involve critical steps such as introducing the precursors in solid form during the reaction or high-temperature injection. These critical steps determine the subsequent course of the reaction, and a small mistake or change in parameters can result in losing control over the nanoparticles’ growth and, simultaneously, over the optical and structural characteristics of the end product. We show that by varying the Zn(Ac)_2_ concentration while keeping the other reaction parameters constant, one can obtain CdS dots, rods, or platelets in the heating-up synthesis. The proposed synthesis route is straightforward and allows for the obtention of crystals with different confinement types (0D, 1D, and 2D structures) using the same method and varying only the amount of one reagent added to the initial solution as illustrated in Figure 1. Moreover, we investigated the platelets’ formation via optical spectroscopy and structural characterization and compared our results with other groups’ reports. In addition, we observed high-temperature, magic-size clusters formation during the initial synthesis stage.

## 2. Materials and Methods 

### 2.1. Materials

Cadmium oxide (CdO) (99.5%), oleic acid (OA) (technical grade 90%), octadecene (ODE) (technical grade 90%), zinc acetate (Zn(Ac)_2_) (99.99%), and sulfur (99.998%) were purchased from Sigma–Aldrich (now Merck KGaA, Darmstadt, German); ethanol (analytical grade 99.8%) and isopropanol (analytical grade 99.7%) from Chempur (Piekary Slaskie, Poland); acetone (100.0%) from VWR International Sp. z o.o. (Gdansk, Poland); hexane (99%) from Avantor Performance Materials Poland S.A. (Gliwice, Poland). All chemicals were used without further purification. 

### 2.2. Cadmium Precursor—Cd(OL)_2_

1027.3 mg (8 mmol) of CdO, 6.880 mL (21.8 mmol) of OA, and 13.120 mL (41 mmol) of ODE were mixed in a three-neck flask, degassed for 20 min, and heated to 250 °C until the formation of a clear colorless solution. After the reaction the mixture was degassed for 20 min.

### 2.3. Sulphur Precursor—S-ODE

64.1 mg (2 mmol) of S and 10 mL (31.3 mmol) of ODE were mixed in a three-neck flask and degassed. The solution was heated to 140 °C until a clear liquid was formed.

### 2.4. CdS Nanocrystals Synthesis

1 mL of Cd(OL)_2_, 1 mL of S-ODE, 18 mL of ODE, and various amounts of Zn(Ac)_2_ (from 0 to 0.5 mmol) were mixed in a three-neck flask and degassed for 20 min. The reaction temperature was then set to a value between 180 °C and 230 °C and maintained for 60 or 120 min. Aliquots were taken from the reaction mixture at 1, 2, 4, 6, 9, 15, 30, and 60 min to monitor the crystal growth. For the syntheses conducted at 180 °C, a control sample was also taken when the temperature reached 130 °C, and for the higher temperature syntheses, additional aliquots were collected at 180 °C, 200 °C, 220 °C, and 230 °C.

### 2.5. Nanocrystals Purification

After the synthesis nanocrystals were purified using one of the two methods:

(1) A mixture of isopropanol and acetone was added to the synthesis solution, which was then centrifuged at 6000 rpm (4430 rcf) for 10 min. The supernatant was decanted, and the precipitate was redispersed in hexane. The procedure was then repeated.
(2) The synthesis solution with the addition of a small amount of OA was centrifuged at 6000 rpm (4430 rcf) for 10 min. The precipitate was collected and redispersed in hexane. The nanocrystals were precipitated again with ethanol, centrifuged, and redispersed. The second step was repeated. This procedure allowed for the removal of quantum dots, which can appear as a side product of the platelets’ synthesis, from the solution.

### 2.6. Optical Spectroscopy Measurements

All absorbance (ABS) spectra were recorded by a JASCO V-570 spectrophotometer (JASCO International Co., Ltd., Tokyo, Japan). Photoluminescence (PL) spectra were collected using FL 8500 Fluorescence Spectrophotometer (PerkinElmer Inc., Waltham, MA, USA) with a Xenon lamp as the excitation source. The excitation wavelength was selected to be 300 nm via monochromator. 

### 2.7. Structural Measurements

FEI TITAN3 G2 60-300 transmission electron microscopy (TEM, Thermo Fisher Scientific, Hillsboro, OR, USA) was applied to record nanocrystals images, while energy-dispersive X-ray spectroscopy (EDX, Thermo Fisher Scientific, Hillsboro, OR, USA) was applied to determine the nanocrystals’ composition. Samples for experiments were prepared by the evaporation of diluted solutions of purified nanocrystals on carbon-coated copper grids.

## 3. Results and Discussion

The Zn(Ac)_2_ reagent in one-pot synthesis of CdS quantum dots has previously been used by Banski et al. [21] to enhance the nanocrystals photoluminescence stability. Adding a small amount of Zn(Ac)_2_ resulted in the incorporation of zinc ions into the CdS nanocrystal forming a CdS core/CdZnS thin alloy shell structure. Acetate ligands have also been proven to cover the crystal surface along with oleate ligands which, together with the thin shell, led to a significant improvement in the optical properties. The emission from mid-gap trap states was diminished, and excellent photoluminescence temporal stability at an excitation power up to 36 mW was observed. Herein, we describe a similar synthesis procedure as in the abovementioned work, namely, a heating-up one-pot reaction of the mixture composed of ODE, Cd(OL)_2_, sulfur, and Zn(Ac)_2_. A small concentration of Zn(Ac)2 resulted in highly luminescent CdS/CdZnS quantum dots, whereas higher concentrations led to the formation of nanoplatelets.

**Figure 2:**
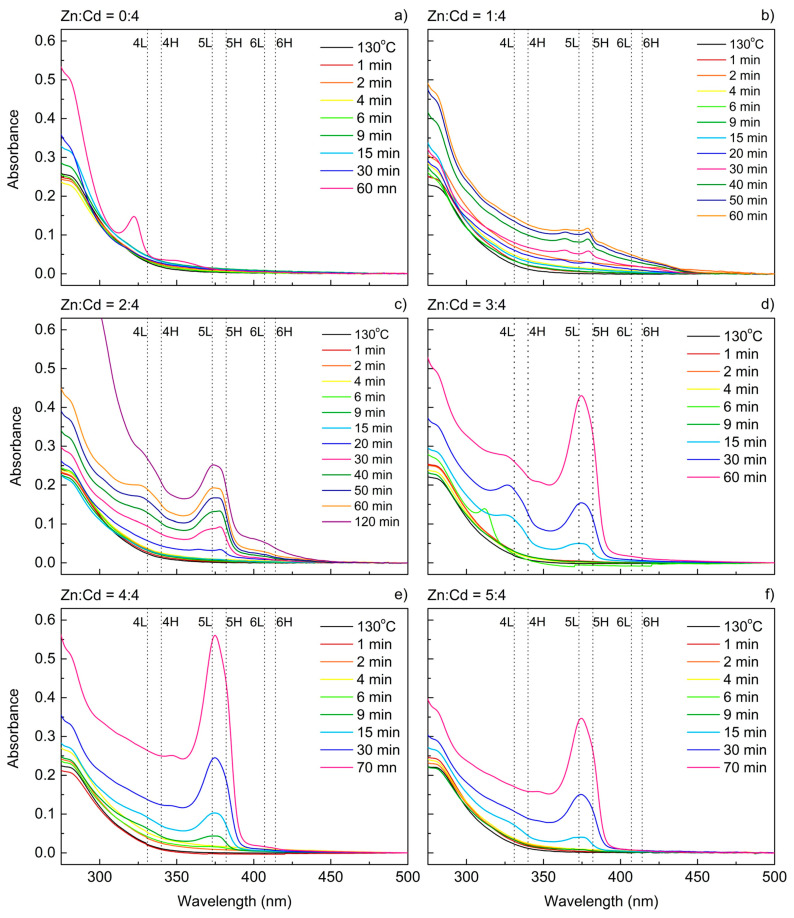
Absorbance spectra of cadmium sulfide (CdS) nanocrystals obtained with different Zn(Ac)_2_ concentrations recorded for aliquots taken at different stages of the reaction. The Zn:Cd ratio is indicated on top of every graph: (**a**) Zn:Cd = 0:4; (**b**) Zn:Cd = 1:4; (**c**) Zn:Cd = 2:4; (**d**) Zn:Cd = 3:4; (**e**) Zn:Cd = 4:4; (**f**) Zn:Cd = 5:4. The reaction temperature for all samples was 180 °C except 130 °C for the first aliquots. The electron-light hole (L) and electron-heavy hole (H) transitions for 4, 5, and 6 ML platelets are marked with dotted lines based on Reference [22].

The results obtained for syntheses with different Zn(Ac)_2_ quantities are presented in Figure 2. All the other reaction parameters (i.e., S and Cd(OL)_2_ concentrations) were fixed, with the temperature set at 180 °C. For no zinc added (Figure 2a), the appearance of any absorbance (ABS) features was not visible for 30 min of the reaction. However, the ABS spectrum recorded after 60 min clearly indicates, that the synthesis result were quantum dots, as the peak, situated at 323 nm, did not match either of the transitions typical for the CdS platelets. The electron-light hole (L) and electron-heavy hole (H) transitions for CdS nanoplatelets were compared with the literature [22] and were in good agreement with our results. The absorbance peak at 323 nm suggest the presence of CdS magic-size clusters as reported by Yu et al. [23,24]. The clusters’ size can be estimated based on the work of Yu et al. [25] to be ~1.6 nm.

For the molar ratio for Zn:Cd of 1:4 (Figure 2b), the absorbance spectrum still did not manifest a NPL-like appearance, but the maxima remained very close to the electron-light hole and electron-heavy hole transitions for five monolayer (ML)-thick NPLs, which are marked in the graph as 5L and 5H, respectively. It should be noted that in this convention, a 5 ML thick platelet consists of five layers of S and six layers of Cd in accordance with Riedinger et al. [20]. Starting from a Zn:Cd ratio of 2:4 (Figure 2c), the ABS spectra resembled proper NPLs. For both 1:4 and 2:4, the ABS features started to arise after 20 min of synthesis reaction. In the 2:4 sample, the population of 4 and 5 ML can be found, whereas the presence of 6 ML NPLs is questionable. A further increase in the Zn concentration (Figure 2d–f) leads to faster growth rates and suppresses the 4 ML thick platelets’ formation. For the Zn:Cd ratio of 3:4, the 4 ML population was still visible at the end of the reaction (60 min), but for 4:4 and 5:4, it disappeared completely by this time. It should be noted that the given reaction conditions allow to obtain purely one population of NPLs—5 ML for those two samples, which can be hard to achieve in the case of one-pot reactions, as they usually produce several NPLs populations with different thicknesses. For this reason, the one-pot approach is rarely used, whereas the majority of the methods reported in the literature use multi-step reactions involving the addition of reagents (mostly pouring of powder precursors) at a precisely defined moment. Since the addition step is crucial, slight variations of the addition manner and time affect the final result thus making this synthesis method poorly reproducible. This problem was solved in our approach.

A very interesting ABS spectrum was recorded in the middle range of Zn(Ac)_2_ concentration (Zn:Cd = 3:4) after 6 min of the synthesis. It contained a single peak at 312 nm, which can be attributed to the already reported CdS MSCs [23,24,26]. We can therefore speculate that the intermediate product in the platelets’ formation were small (~1.5 nm [25]) CdS clusters, which would agree well with the work of Wang et al. [18,19] concerning CdSe, CdS, ZnS, ZnS, and CdTe NPLs. Nevertheless, formation of MSCs at high-temperature NPLs synthesis was not reported so far to the best of our knowledge.

**Figure 3:**
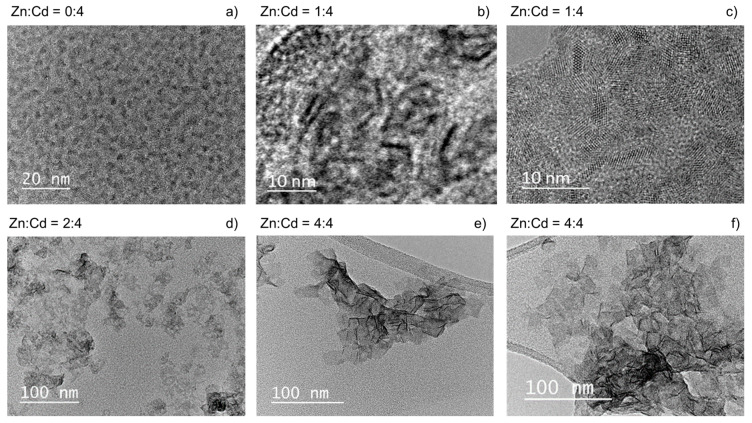
TEM images of four CdS nanocrystals obtained with different Zn(Ac)_2_ concentrations. The Zn:Cd ratio was (**a**) 0:4, (**b**,**c**) 1:4, (**d**) 2:4, and (**e**,**f**) 4:4. The reaction temperature for all samples was 180 °C, and the reaction time—60 min.

To directly analyze the shape of the obtained nanocrystals, the samples were analyzed via transmission electron microscopy (TEM). Figure 3 shows TEM images of nanocrystals synthesized at 180 °C for 60 min with Zn:Cd molar ratios of 0:4, 1:4, 2:4, and 4:4. The reaction without zinc acetate resulted in isotropic shapes of the particles with average diameters below 2 nm (Figure 3a), which confirms assumptions made based on absorbance spectra (Figure 3a). In the case of the 1:4 ratio, the crystals took the form of elongated structures that were not yet quasi two-dimensional (Figure 3b,c). The sample was not homogenous, but the oblong shape was clearly visible. A further increase in the Zn(Ac)_2_ concentration (ratio 2:4) resulted in a mixture of different shapes: dots, rods, and irregular platelets (Figure 3d). For the largest Zn(Ac)_2_ amount (ratio 4:4), a clear platelet-like shape was observed on the TEM image which, in accordance with optical studies, confirms the successful synthesis of 2D NPLs in heating-up synthesis (Figure 3e,f). For better visibility the sample was precipitated using purification method 2 (see Section 2, Nanocrystal purification), which allowed the removal of the dot-shaped side product of the reaction. However, the samples with optical features visibly different from the ABS spectra of 2D-shaped nanocrystals (Zn:Cd molar ratios of 0:4, 1:4, and 2:4) were treated with the standard purification method (denoted as 1 in the description) to ensure that all smaller particles would be precipitated as well. An example of TEM images for samples treated with method 1 and 2 are shown in Figure S1.

The EDX spectroscopy was applied to verify the presence of Zn elements in nanocrystals. An example spectrum, which was recorded at a 4:4 Zn:Cd molar ratio, is presented in Figure S2 in the Supplementary Materials. No peaks related to Zn could be distinguished in the spectrum, suggesting that even at the highest Zn precursor content the final NPLs were composed only of CdS. Thus, Zn(Ac)_2_ did not incorporate into the NPLs’ structure and did not participate in the crystals’ growth directly; however, it was crucial for the anisotropic growth of the cubic phase of the CdS NPLs. As stated by Riedinger et al. [20], the precursor solubility can trigger phase separation and 2D growth, but in this case, Zn(Ac)_2_ did not serve as a precursor at all. It might, however, create some kinds of nucleation centers surrounding the cadmium monomers, preventing them from falling apart and, thus, shifting the critical barrier of nucleation and affecting the growth mode. The Zn(Ac)_2_ compound could be responsible for the appearance of another phase in the mixture in which the Cd ions accumulate and undergo a growth process with surface-reaction-limited kinetics. It has been shown that various acetate compounds may serve a similar role. Ithurria et al. [27] tested acetate salts including Na(Ac), Mn(Ac)_2_, Mg(Ac)_2_, Co(Ac)_2_, and Zn(Ac)_2_ in the CdSe NPLs’ synthesis, and all of them allowed to obtain flat 2D structures, which suggests that the short carboxylic acetate chain is the shape-defining factor rather than the Zn cation. We chose the Zn(Ac)_2_ compound over other salts, because in our previous work, we observed that it improves the luminescent properties of QDs [21], whereas, for example, Cd(Ac)_2_ did not give the same effects. 

**Figure 4:**
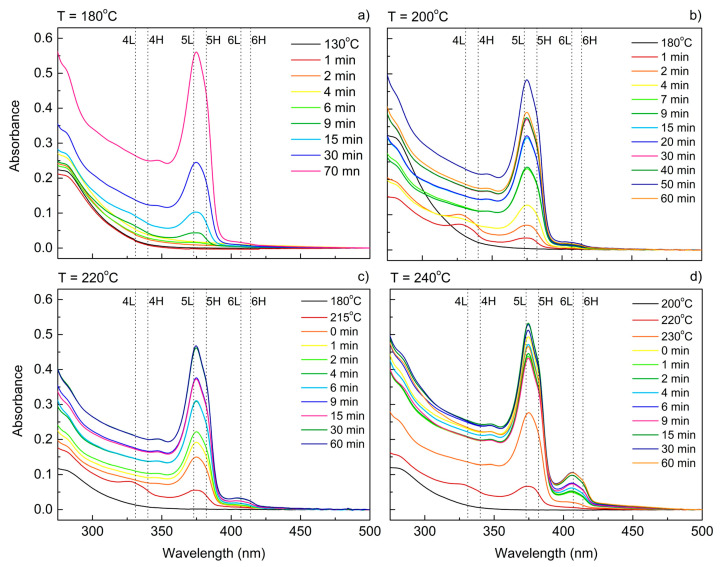
Absorbance spectra of CdS nanocrystals obtained with different reaction temperatures and fixed Zn(Ac)_2_ concentration recorded for aliquots taken at different stages of the reaction. The reaction temperature is indicated on top of every graph. The Zn:Cd ratio for all samples was 4:4. The electron-light hole (L) and electron-heavy hole (H) transitions for 4, 5, and 6 ML platelets are marked with dotted lines based on Reference [22]. The reaction temperatures are: (**a**) 180 °C; (**b**) 200 °C; (**c**) 220 °C; (**d**) 240 °C.

**Figure 5:**
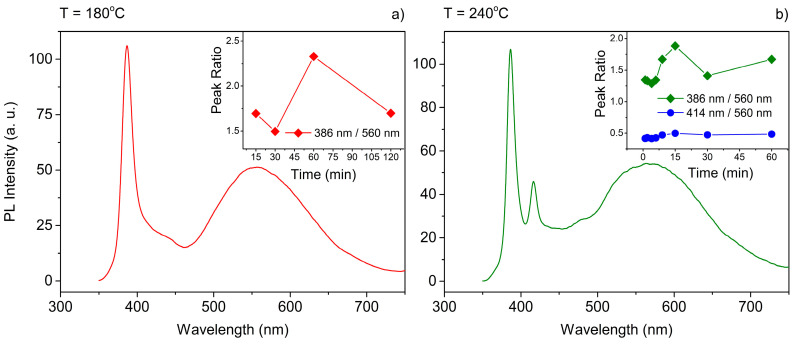
Photoluminescence spectra of CdS nanocrystals obtained with different reaction temperatures and fixed Zn(Ac)_2_ concentration. The insets show the ratio of the peak intensity related with excitonic emission to the peak related with defects for those spectra. The reaction temperature is indicated on top of every graph: (**a**) 180 °C; (**b**) 240 °C. The Zn:Cd ratio for all samples was 4:4.

In order to further investigate the mechanism of the NPLs’ growth, the influence of the reaction temperature was studied. The solubility of all reagents is highly dependent on the mixture temperature and, as was shown previously, it serves a crucial role in defining the reaction kinetics. Thus, the temperature factor should be able to serve a similar role to the short-chained precursor concentration. For the set of syntheses with mixtures containing a specified precursors composition (Zn:Cd molar ratio of 4:4), the synthesis temperature was changed in the range of 180 to 240 °C, while other parameters were kept constant. Beginning from 200 °C, the 6 ML population started to appear, as was evident from the peak at ~415 nm in the ABS spectra (Figure 4b–d). The intensity of this peak increased with the temperature and was the highest at 240 °C, suggesting that the concentration of 6 ML CdS NPLs increased when increasing the synthesis temperature. In higher temperatures, the emergence of NPLs having 4 ML as well as 5 ML was also more rapid; they appeared almost immediately when the temperature exceeds 200 °C. For NPL samples grown at 180 °C and 240 °C, the photoluminescence (PL) spectra were also recorded (Figure 5). For the 180 °C synthesis (Figure 5a), the maximum of excitonic emission from 5 ML platelets was at 386 nm and the maximum of excitonic emission from 5 ML platelets at 386 nm, which corresponds to very small Stokes shifts (4 nm).

A broad band on the long-wavelength side indicates the existence of defect states. In the case of NPLs synthesized at 240 °C (Figure 5b), additional emission bands from the 6 ML population are visible with the maximum at 416 nm and an even smaller Stokes shift (2 nm) was determined for it. In the insets of Figure 5a,b, the ratios of the peak intensity (relative peak heights) related to excitonic emission and defect state emission are shown. This intensity ratios were analyzed in various stages of the synthesis to determine the optimal time for maximizing the excitonic emission. During synthesis at 180 °C the emission intensity from defect states decreased during the synthesis until about 60 min. Further increasing of the reaction time gave no better results (Figure 5a). For the synthesis temperature 240 °C, we observed two bands related with excitonic emission and both of them were analyzed in terms of emission intensity compared to defect states emission. The intensity of excitonic photoluminescence coming from 5 ML CdS NPLs (386 nm) increased gradually in relation to defect-related transitions within 15 min of the reaction when it reached minimum value. Then, the defect-related emission became more intensive; however, after 30 min the excitonic emission started to get stronger again. As for the 6 ML transition (416 nm), the emission was always weak, and the peak was approximately two times lower than the defect-related emission. The overall trend was similar to 5 ML, but the changes were less significant. 

TEM observations confirm the flat shape of obtained nanostructures (Figure S3). Similar to samples synthesized at lower temperatures and high Zn(Ac)_2_ concentrations (Figure 2e,f), the NPLs grown at 240 °C have irregular, roughly rectangular shape and are accompanied by dot-like structures.

## 4. Conclusions

We presented a complex study of heating-up one-pot synthesis of CdS NPLs using a Zn(Ac)_2_ as a trigger for the shape-controlled growth. Tuning the morphology of CdS nanocrystals and changing their shape from 0D to 2D, was obtained just by varying the concentration of Zn(Ac)_2_ used in the synthesis. We showed that a small amount of acetate compound is already sufficient to induce the shape anisotropy. A minor change in the solution composition during the initial solution preparation serves as a very simple morphology controlling factor in opposition to complicated reactions with critical steps such as high-temperature injection of precursors described in the literature. The Zn(Ac)_2_ does not serve as a precursor and is not incorporated in the nanocrystals structure, but it affects the reaction kinetics. We postulate that it surrounds the cadmium monomers and creates phase-separated centers for the crystal nucleation. The obtained results allowed us to define the synthesis conditions at which a purely 5 mL population of flat, luminescent CdS NPLs, as well as elongated rods or small isotropic dots can be effectively synthesized using a convenient and repeatable one-pot approach. The possible growth mechanism, involving magic-size clusters as a transition stage for 2D platelets formation was discussed as well, since for the first time, we observed MSCs at a high temperature. They exist at the beginning of the synthesis, as confirmed via optical measurements, and disappeared later on being incorporated into the NPLs’ structure.

## Data Availability

The data presented in this study are available on request from the corresponding author.

## Supplementary Materials

The following are available online at https://www.mdpi.com/1996-1944/14/3/476/s1, Figure S1: Comparison of purification methods, Figure S2: EDX spectra, Figure S3: TEM images, Figure S4: TEM image and size histogram of CdS nanocrystals.

## References

[1] Murray C.B., Norris D.J., Bawendi M.G. (1993). Synthesis and Characterization of Nearly Monodisperse CdE (E = S, Se, Te) Semiconductor Nanocrystallites. J. Am. Chem. Soc..

[2] Song Q., Zhang Z.J. (2004). Shape Control and Associated Magnetic Properties of Spinel Cobalt Ferrite Nanocrystals. J. Am. Chem. Soc..

[3] Cheon J., Kang N.J., Lee S.M., Lee J.H., Yoon J.H., Oh S.J. (2004). Shape Evolution of Single-Crystalline Iron Oxide Nanocrystals. J. Am. Chem. Soc..

[4] Carbone L., Nobile C., De Giorgi M., Della Sala F., Morello G., Pompa P., Hytch M., Snoeck E., Fiore A., Franchini I.R. (2007). Synthesis and micrometer-scale assembly of colloidal CdSe/CdS nanorods prepared by a seeded growth approach. Nano Lett..

[5] Grebinski J.W., Hull K.L., Zhang J., Kosel T.H., Kuno M. (2004). Solution-based straight and branched CdSe nanowires. Chem. Mater..

[6] Nasilowski M., Mahler B., Lhuillier E., Ithurria S., Dubertret B. (2016). Two-Dimensional Colloidal Nanocrystals. Chem. Rev..

[7] Kumar S., Nann T. (2006). Shape Control of II–VI Semiconductor Nanomaterials. Small.

[8] Hughes B.K., Luther J.M., Beard M.C. (2021). The Subtle Chemistry of Colloidal, Quantum-Confined Semiconductor Nanostructures. ACS Nano.

[9] Manna L., Wang L.W., Cingolani R., Alivisatos A.P. (2005). First-principles modeling of unpassivated and surfactant-passivated bulk facets of wurtzite CdSe: A model system for studying the anisotropic growth of CdSe nanocrystals. J. Phys. Chem. B.

[10] Zhang F., Wang S., Wang L., Lin Q., Shen H., Cao W., Yang C., Wang H., Yu L., Du Z. (2016). Super color purity green quantum dot light-emitting diodes fabricated by using CdSe/CdS nanoplatelets. Nanoscale.

[11] Chen Z., Nadal B., Mahler B., Aubin H., Dubertret B. (2014). Quasi-2D Colloidal Semiconductor Nanoplatelets for Narrow Electroluminescence. Adv. Funct. Mater..

[12] Guzelturk B., Kelestemur Y., Olutas M., Delikanli S., Demir H.V. (2014). Amplified spontaneous emission and lasing in colloidal nanoplatelets. ACS Nano.

[13] She C., Fedin I., Dolzhnikov D.S., Dahlberg P.D., Engel G.S., Schaller R.D., Talapin D.V. (2015). Red, Yellow, Green, and Blue Amplified Spontaneous Emission and Lasing Using Colloidal CdSe Nanoplatelets. ACS Nano.

[14] Li M., Zhi M., Zhu H., Wu W.-Y., Xu Q.-H., Jhon M.H., Chan Y. (2015). Ultralow-threshold multiphoton-pumped lasing from colloidal nanoplatelets in solution. Nat. Commun..

[15] Diroll B.T., Talapin D.V., Schaller R.D. (2017). Violet-to-Blue Gain and Lasing from Colloidal CdS Nanoplatelets: Low-Threshold Stimulated Emission despite Low Photoluminescence Quantum Yield. ACS Photonics.

[16] Grim J.Q., Christodoulou S., Di Stasio F., Krahne R., Cingolani R., Manna L., Moreels I. (2014). Continuous-wave biexciton lasing at room temperature using solution-processed quantum wells. Nat. Nanotechnol..

[17] Yang Z., Pelton M., Fedin I., Talapin D.V., Waks E. (2017). A room temperature continuous-wave nanolaser using colloidal quantum wells. Nat. Commun..

[18] Wang Y., Zhang Y., Wang F., Giblin D.E., Hoy J., Rohrs H.W., Loomis R.A., Buhro W.E. (2014). The Magic-Size Nanocluster (CdSe) _34_ as a Low-Temperature Nucleant for Cadmium Selenide Nanocrystals; Room-Temperature Growth of Crystalline Quantum Platelets. Chem. Mater..

[19] Wang Y., Zhou Y., Zhang Y., Buhro W.E. (2015). Magic-size II-vi nanoclusters as synthons for flat colloidal nanocrystals. Inorg. Chem..

[20] Riedinger A., Ott F.D., Mule A., Mazzotti S., Knüsel P.N., Kress S.J.P., Prins F., Erwin S.C., Norris D.J. (2017). An intrinsic growth instability in isotropic materials leads to quasi-two-dimensional nanoplatelets. Nat. Mater..

[21] Banski M., Chrzanowski M., Zatryb G., Misiewicz J., Podhorodecki A. (2018). Enhanced photoluminescence stability of CdS nanocrystals through a zinc acetate reagent. RSC Adv..

[22] Ithurria S., Tessier M.D., Mahler B., Lobo R.P.S.M., Dubertret B., Efros A.L. (2011). Colloidal nanoplatelets with two-dimensional electronic structure. Nat. Mater..

[23] Yu Q., Liu C.Y. (2009). Study of magic-size-cluster mediated formation of cds nanocrystals: Properties of the magic-size clusters and mechanism implication. J. Phys. Chem. C.

[24] Yu Q., Liu C., Zhang Z., Liu Y. (2008). Facile synthesis of semiconductor and noble metal nanocrystals in high-boiling two-phase liquid/liquid systems. J. Phys. Chem. C.

[25] Yu W.W., Qu L., Guo W., Peng X. (2003). Experimental Determination of the Extinction Coefficient of CdTe, CdSe, and CdS Nanocrystals. Chem. Mater..

[26] Pan D., Jiang S., An L., Jiang B. (2004). Controllable synthesis of highly luminescent and monodisperse CdS nanocrystals by a two-phase approach under mild conditions. Adv. Mater..

[27] Ithurria S., Dubertret B. (2008). Quasi 2D colloidal CdSe platelets with thicknesses controlled at the atomic level. J. Am. Chem. Soc..

