# Supplementary material for: CdS Dots, Rods and Platelets—How to Obtain Predefined Shapes in a One-Pot Synthesis of Nanoparticles
Source: Materials (Basel). 2021 Jan 20;14(3):476. doi: 10.3390/ma14030476 (PMC7864161; doi:10.3390/ma14030476)
Supplement: Supplementary file 1 [file materials-14-00476-s001.pdf]

Supplementary material

CdS Dots, Rods, and Platelets—How to Obtain Predefined Shapes in a One-Pot Synthesis of Nanoparticles

Hanna Woznica, Mateusz Banski * and Artur Podhorodecki

Comparison of the purification methods.

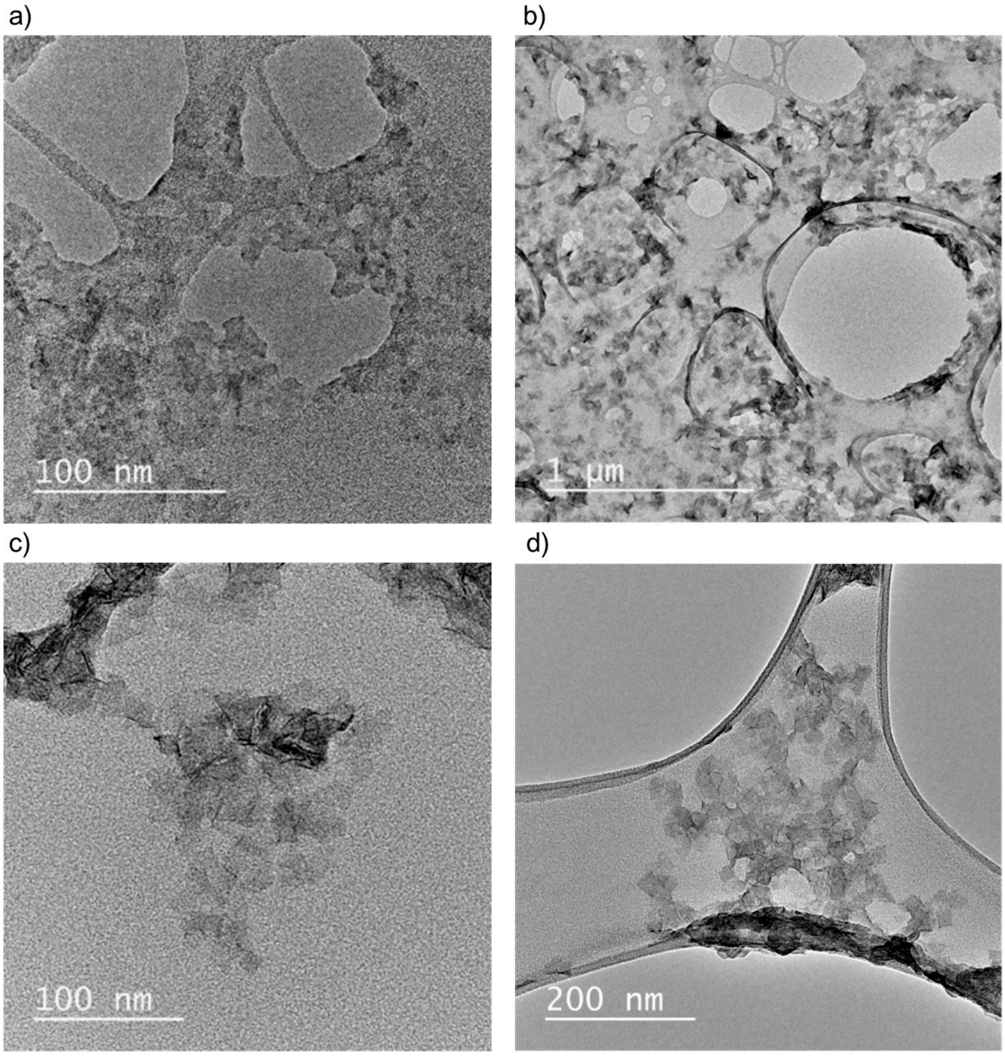

Figure S1. TEM images of nanocrystals purified with method 1) (**a**, **b**) and with method 2) (**c**, **d**). For method 1) side products of the reaction—quantum dots – are still wisible. Samples treated with method 2) show no trace of dots.

EDX spectra.

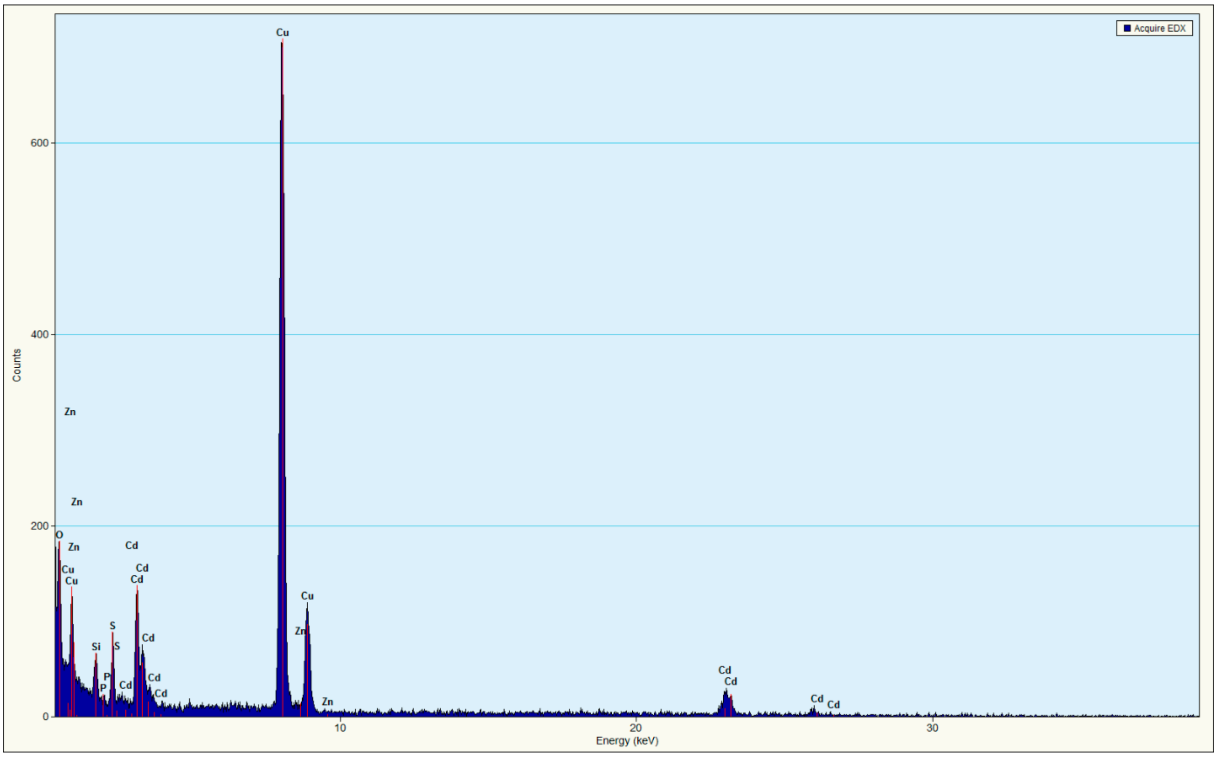

Figure S2. EDX spectrum obtained for the Zn:Cd ratio 4:4 and temperature 180 °C. The spectra indicates that no Zn is present in the sample. Cu comes from the support grid used in TEM measurements. The area from which the spectrum was collected is showed above.

TEM images.

T = 240°C

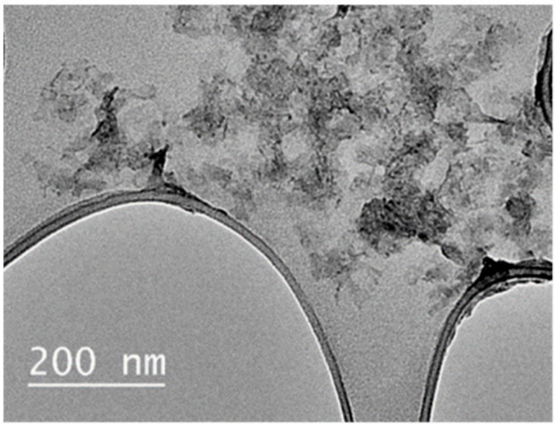

Figure S3. TEM image for the sample synthesized in 240 °C with Zn:Cd molar ratio 4:4. The nanocrystals have irregular, flat shape and are accompanied by dot-like structures.

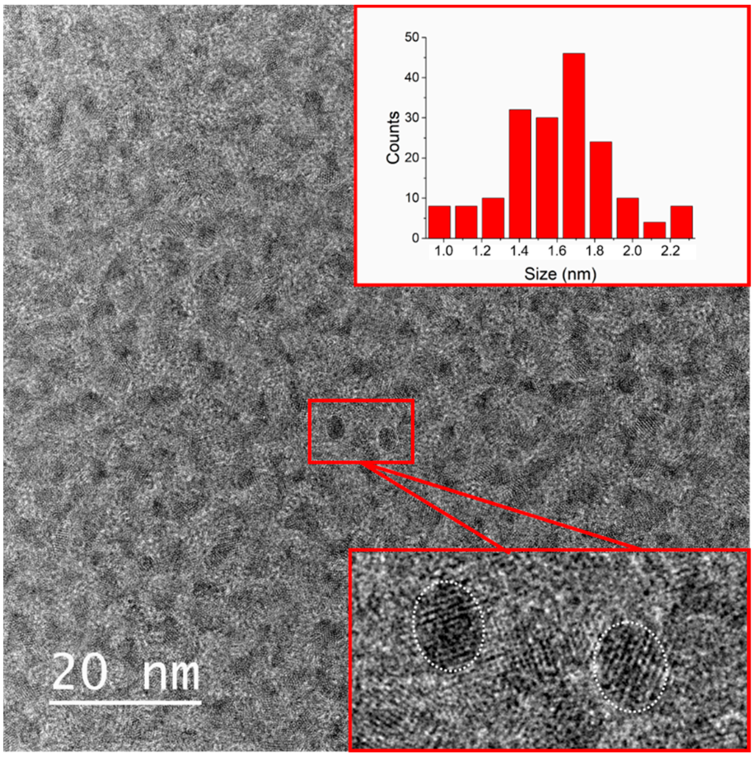

Figure S4. TEM image of CdS nanocrystals obtained with Zn:Cd = 0:4 ratio (no zinc added) and reaction temperature 180 °C. The insets show a size distribution and an example of extracting the nanocrystals boundaries.
